# Meningitis With Staphylococcus aureus Bacteremia in an Older Patient With Nonspecific Symptoms: A Case Report

**DOI:** 10.7759/cureus.34153

**Published:** 2023-01-24

**Authors:** Airi Minatogawa, Junya Ohara, Yuta Horinishi, Chiaki Sano, Ryuichi Ohta

**Affiliations:** 1 Family Medicine, International University of Health and Welfare, Tokyo, JPN; 2 Family Medicine, Matsue Seikyo Hospital, Matsue, JPN; 3 Community Care, Unnan City Hospital, Unnan, JPN; 4 Community Medicine Management, Shimane University Faculty of Medicine, Izumo, JPN

**Keywords:** erector pili muscle abscess, meningitis, staphylococcus aureus bacteremia, general malaise, rural hospital, general medicine

## Abstract

*Staphylococcus aureus* bacteremia has been associated with various symptoms that can spread to diverse organs, including the meninges, which can be challenging to diagnose given the nonspecific symptoms. Early examination, including assessment of the cerebrospinal fluid, is necessary when a patient is diagnosed with *S. aureus* bacteremia accompanied by unconsciousness. A 73-year-old male presented to our hospital complaining of general malaise without fever. The patient developed impaired consciousness immediately after hospitalization. Following the investigations, the patient was diagnosed with *S. aureus* bacteremia and meningitis. If a patient presents with symptoms of unknown cause and acute progressive disease, meningitis and bacteremia should always be considered. Blood cultures should be performed promptly, affording an early diagnosis, bacteremia treatment, and the establishment of meningitis.

## Introduction

Staphylococcus aureus can infect diverse organs, and diagnosis is often challenging, especially in immunosuppressed patients, who frequently exhibit nonspecific symptoms, such as fever and malaise [1]. Considering meningeal, cardiac, or muscle infections, establishing S. aureus infection by blood culture assessment may be the basis for an in-depth examination [2-4], although symptoms may have progressed gradually. The possibility of S. aureus infection should always be raised early in immunosuppressed patients presenting various complaints [5].

Meningitis and intramuscular abscesses caused by S. aureus are hematogenous bacterial infections that are more common in tropical regions; however, their frequency is growing in temperate regions and is associated with underlying diseases, such as human immunodeficiency virus (HIV) infection and other immunodeficiency disorders, as well as drug injections and malnutrition [6]. Tropical pyomyositis mainly impacts children aged two to five years and adults aged 20 to 45, whereas the temperate climate variant primarily occurs in adult males with comorbidities [7]. A 73-year-old male subject presented to our hospital with complaints of general malaise, without fever. The patient developed impaired consciousness immediately after hospitalization. After relevant investigations, a diagnosis of S. aureus bacteremia and meningitis was achieved. Blood culture results for meningitis revealed S. aureus bacteremia, which established the presence of a deep muscle abscess. The present case demonstrates the effectiveness of promptly investigating and treating vague symptoms in elderly patients, implying the crucial implications for future medical care among geriatric patients.

## Case presentation

A 73-year-old male subject presented to a rural community hospital with chief complaints of hematuria, back pain, and anorexia. He had yellowish-brown urine for one week. On the day before the hospital visit, he experienced hematuria and back pain. On the day of admission, he experienced restricted mobility, prompting his visit to our hospital. He had a history of dyslipidemia and hyperuricemia. The patient was on pemafibrate, atorvastatin, and febuxostat.

On arrival, vital signs were as follows: temperature, 37.2°C; heart rate, 100/min; blood pressure, 98/63 mmHg; respiratory rate, 18/min; oxygen saturation (SpO2), 98% (ambient air); consciousness, normal. Physical examination revealed an icterus of the eyes and a scaly skin rash on the left upper extremity. The lung, heart, and neurological examination results were all normal. Laboratory tests revealed elevated hepatobiliary enzyme levels, hypercalcemia, and increased creatinine kinase levels (Table 1).

**Table 1:** Initial patient laboratory data

Parameters	Level	Reference
White blood cells	8.20	3.5–9.8 × 10^3^/μL
Neutrophils	85.3	44.0–72.0 %
Lymphocytes	5.8	18.0–59.0 %
Monocytes	8.6	0.0–12.0 %
Eosinophil	0.0	0.0–10.0 %
Basophils	0.3	0.0–3.0 %
Red blood cells	4.40	4.10–5.30 × 10^6^/μL
Hemoglobin	10.9	13.5–17.6 g/dL
Hematocrit	32.8	36–48 %
Mean corpuscular volume	74.7	82–101 fl
Platelets	27.8	13.0–36.9 × 10^4^/μL
Total protein	7.2	6.6–8.1 g/dL
Albumin	2.0	3.9–4.9 g/dL
Total bilirubin	2.6	0.2–1.2 mg/dL
Direct bilirubin	1.7	0.03–0.40 mg/dL
Aspartate aminotransferase	82	8–38 IU/L
Alanine aminotransferase	86	4–44 IU/L
γ-Glutamyl transpeptidase	165	16–73 IU/L
Lactate dehydrogenase	211	106–211 U/L
Blood urea nitrogen	22.9	8.0–20.0 mg/dL
Creatinine	0.88	0.40–1.10 mg/dL
Estimated glomerular filtration rate	65.1	>60.0 mL/min/L
Serum Na	126	135–147 mEq/L
Serum K	3.8	3.3–4.8 mEq/L
Serum Cl	88	98–108 mEq/L
Serum Ca	12.1	8.8–10.2 mEq/L
Serum P	2.1	2.7–4.6 mEq/L
Serum Mg	1.8	1.8–2.3 mEq/L
Creatine phosphokinase	323	56–244 U/L
Thyroid-stimulating hormone	0.77	0.35–4.94 μIU/mL
Free T3	<1	1.88-3.18 ng/dL
Free T4	1.0	0.70–1.48 ng/dL
Immunoglobulin G	961	870–1700 mg/dL
Immunoglobulin M	95	35–220 mg/dL
Immunoglobulin A	308	110–410 mg/dL
Urine protein	Negative	Negative
Urine blood	3+	Negative

We considered the possibility of occult bacteremia and performed blood culture tests with an appropriate method. On the second day of hospitalization, the patient had a fever of 38.9°C and decreased oxygenation from the morning, and a physical examination revealed neck rigidity. Assessment of the cerebrospinal fluid (CSF) revealed decreased glucose levels and increased cell counts (protein 750 mg/dL, glucose 25 mg/dL, cell count 96 /µL (100% neutrophil), the negative antigen of Streptococcus pneumoniae in the CSF (-)) without no growth in bacteria culture. The patient was unconscious, and a simple computed tomography (CT) scan of the head was performed to examine intracranial lesions; however, no intracranial lesions were detected. To address bacterial meningitis, we initiated treatment with meropenem 6 g/day, vancomycin 2 g/day, acyclovir 2250 mg/day, and ceftriaxone 2 g/day. 　

A blood culture test on the third day of hospitalization revealed the presence of gram-positive cocci, and cefazolin 6 g/day was administered. Subsequently, transthoracic echocardiography was performed to establish potential infective endocarditis (IE), but no obvious verrucae were observed without any valvular lesions. Because of the lack of any valvular lesions, we did not perform transesophageal echocardiography, considering the low risk of IE. Magnetic resonance imaging (MRI) of the head showed acute diffuse brainstem encephalitis as a possible complication. Two days later, a blood culture revealed methicillin-susceptible S. aureus (MSSA). Before admission, erythema with bedsores and pruritus were observed, and bacteremia due to compromised skin barrier function was considered.

**Figure 1:**
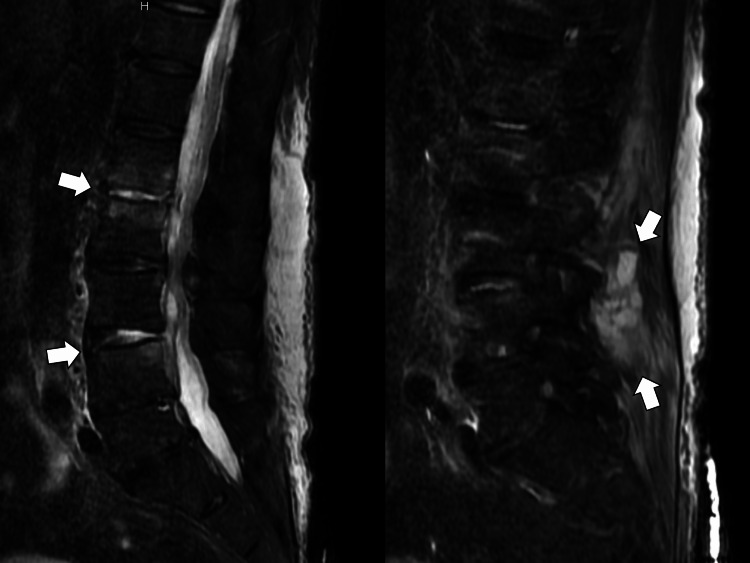
Magnetic resonance imaging (short tau inversion recovery) showing multiple vertebritis and paraspinal abscesses (white arrows)

On the sixth day of hospitalization, the patient complained of low back pain, and the physical examination revealed lumbar tapping pain. An MRI (short tau inversion recovery) of the lumbar spine was performed for a close examination, and multiple vertebritis/paravertebral abscesses were diagnosed (Figure 1).

Given that vital signs remained unaltered, treatment with antimicrobial agents was continued. On the 16th day of hospitalization, the patient was switched to minocycline (200 mg/day) based on antimicrobial susceptibility of MSSA. The patient had good clinical course and rehabilitation was continued. On the 30th day of hospitalization, the patient could walk with canes and was transferred to a rehabilitation ward for the preparation of the discharge to his home.

## Discussion

Given the findings of the present case report, it can be suggested that when dealing with multiple complaints in the elderly, it is crucial to consider the possibility of sepsis and bacteremia when the disease course is acute and promptly proceed with a thorough examination. It is necessary to strengthen early treatment protocols by conducting a systematic examination while awaiting culture results.

Elderly patients may experience multiple diverse complaints, and it is necessary to focus on the speed of symptom progression rather than disease specifics [8,9]. Considering the present case, the elderly patient presented with systemic symptoms that worsened weekly, resulting in a diagnosis of meningitis and multiple abscesses against a background of S. aureus bacteremia. Given the progressively worsening symptoms, cultures were obtained to establish the possibility of sepsis or bacteremia. The patient was admitted for follow-up observation, and prompt response to the changes after admission enabled early antimicrobial therapy and treatment of encephalitis symptoms associated with meningitis. Accordingly, the patient was considered to have a favorable outcome. Symptoms in the elderly tend to be more ambiguous owing to physiological changes when compared with those in younger patients [10,11]. Furthermore, administering appropriate medical treatment may present challenges among elderly patients. Therefore, the aging population may experience more severe acute illnesses [12]. Several symptoms in the elderly may resolve spontaneously, potentially contributing to the delayed detection of a trend toward severe disease [13]. Importantly, persistent worsening of symptoms should be carefully noted. In case of persistently worsening complaints in the elderly, prompt investigation should be pursued with the possibility of systemic disease, including bacteremia, at an early stage.

The incidence of S. aureus bacteremia is increasing among the elderly population, necessitating early detection and treatment. In the present case, there were no obvious signs of S. aureus bacteremia before admission. The patient's worsening generalized symptoms suggested the possibility of bacteremia. He was effectively treated based on culture tests and subjected to inpatient follow-up. S. aureus is an epidermal bacterium endemic to the human body [1,5]. Severe infections are rare in patients with a normal immune system. However, when the immune system is weakened by stress or aging, S. aureus can enter the body and induce infections [14]. Furthermore, the possibility of bacteremia increases in the case of a compromised epidermis, which is known to comprise substantial S. aureus bacteria [15]. Considering the present case report, the patient experienced dry skin across the whole body, accompanied by mild bedsores, considered the “gateway for infection.” As frailty progresses, the immune status of the elderly is likely to deteriorate substantially.

In community hospitals with an aging population, it is necessary to respond appropriately to complaints reported by the elderly. When system-specialist general practitioners address complaints of the elderly presenting no progressive signs, symptomatic treatment should carefully consider the patient's perception of symptoms and the surrounding environment that may contribute to symptoms [16]. However, when symptoms are confirmed to be progressive, it is important to consider the possibility of early-stage bacteremia and conduct an in-depth examination, as undertaken in the present case. General physicians need to acquire prompt investigation of unconscious patients comprehensively and improve the health status of older patients in communities [17,18].

## Conclusions

Multiple complaints of S. aureus bacteremia in an elderly patient may lead to persistent exacerbations. When an elderly patient presents with exacerbating multi-organ symptoms, an early diagnosis of S. aureus bacteremia should be considered, and close examination and treatment should be performed. As a general practitioner, it is crucial to consider the progressive nature of symptoms and adjust the speed of treatment to respond smoothly to multiple complaints in the elderly population.
